# Role of post-translational modifications of Sp1 in cancer: state of the art

**DOI:** 10.3389/fcell.2024.1412461

**Published:** 2024-08-20

**Authors:** Xutao Sun, Chengpu Xiao, Xinyang Wang, Siyu Wu, Zhendong Yang, Bowen Sui, Yunjia Song

**Affiliations:** ^1^ Department of Typhoid, School of Basic Medical Sciences, Heilongjiang University of Chinese Medicine, Harbin, China; ^2^ Department of Chinese Internal Medicine, First Affiliated Hospital, Heilongjiang University of Chinese Medicine, Harbin, China; ^3^ Department of Pneumology, First Affiliated Hospital, Heilongjiang University of Chinese Medicine, Harbin, China; ^4^ Department of Pharmacology, School of Basic Medical Sciences, Heilongjiang University of Chinese Medicine, Harbin, China

**Keywords:** Sp1, PTMs, cancer, biomarkers, signaling pathway

## Abstract

Specific protein 1 (Sp1) is central to regulating transcription factor activity and cell signaling pathways. Sp1 is highly associated with the poor prognosis of various cancers; it is considered a non-oncogene addiction gene. The function of Sp1 is complex and contributes to regulating extensive transcriptional activity, apart from maintaining basal transcription. Sp1 activity and stability are affected by post-translational modifications (PTMs), including phosphorylation, ubiquitination, acetylation, glycosylation, and SUMOylation. These modifications help to determine genetic programs that alter the Sp1 structure in different cells and increase or decrease its transcriptional activity and DNA binding stability in response to pathophysiological stimuli. Investigating the PTMs of Sp1 will contribute to a deeper understanding of the mechanism underlying the cell signaling pathway regulating Sp1 stability and the regulatory mechanism by which Sp1 affects cancer progression. Furthermore, it will facilitate the development of new drug targets and biomarkers, thereby elucidating considerable implications in the prevention and treatment of cancer.

## 1 Introduction

Specific protein 1 (Sp1) is a member of the Sp/Kruppel-like factor family; it is expressed in numerous tissues and exhibits variable domains and functions. It consists of three highly homologous type C2H2 zinc fingers in the DNA binding domain that bind to rich GC boxes. It plays a role in controlling bodily functions like cell growth, development, differentiation, and proliferation, as well as embryogenesis, and is also involved in the progression of cancer. Researchers have explored the relationship between Sp1 and cancer, defining the Sp transcription factor as the non-oncogene addiction gene (Hedrick et al., 2016). Research has demonstrated a strong association between Sp1-dependent transcription and a subset of features related to tumor cell development and differentiation. Specifically, Sp1 has been shown to be closely linked to eight “major features” and two “contributing features” within this context. The “major features” encompass cancer cell proliferation, replicative immortalization, resistance to cell death, immune escape, induction of angiogenesis, cell invasion and metastasis, and cell energy disorders, while the “contributing features” include inflammation and unstable genomes (Safe et al., 2023; Kim et al., 2017; Li and Davie, 2010; Beishline; Azizkhan-Clifford et al., 2015; Orzechowska-Licari et al., 2022; Vizcaíno et al., 2015; Hanahan. 2022). Sp1 transcription factors can serve as prognostic indicators in multiple cancers through interaction with miRNA and lncRNA to enhance the growth, viability, movement, and infiltration of cancer cells. Nonetheless, researchers have not developed any anticancer drugs specifically targeting Sp1 for clinical application (Safe. 2023). Post-translational modifications (PTMs) play a pivotal mechanism underlying intracellular regulatory protein function. PTMs of Sp1 protein encompass phosphorylation, ubiquitination, glycosylation, acetylation, SUMOlation, and methylation. These modifications can enhance or inhibit Sp1 protein stability. Additionally, they have the potential to influence the interaction between Sp1 and subsequent elements, impacting the advancement of the cell cycle. (Safe. 2023; Beishline and Azizkhan-Clifford, 2015; Jackson and Tjian, 1988). Therefore, an in-depth examination of the PTMs of Sp1 protein and its impact on cancer diseases will provide novel perspectives for understanding the molecular mechanism underlying cancer incidence and emphasize new objectives and approaches for cancer prevention, detection, and therapy. In this article, we summarize the molecular structure and function of Sp1 protein, in addition to the regulatory mechanism by which PTMs affect cancer initiation and progression.

## 2 Structure and function of Sp1 protein

### 2.1 Structure of Sp1 protein

The human Sp1 protein is a polypeptide chain comprising 785 amino acids with a total molecular weight of 80,693. It is present in various organs, including the brain, kidney, pancreas, lymph, and bone marrow; moreover, it is situated in the cytoplasm and nucleus (Yu et al., 2021). The Sp1 domain is predominantly unstructured, whereas the zinc finger domain is the only structured domain. The Sp1 domain that binds to DNA consists of three consecutive Cys2-His2 zinc finger domains structured in a nuclear magnetic resonance structure (Cook et al., 1999; Oka et al., 2004; Beishline and Azizkhan-Clifford, 2015). The domain controls the basic and responsive gene transcription by detecting the GC cassette recognition element in the promoter of the target gene (Yang et al., 2013). All three zinc finger domains encompass specific sequence preferences, and each zinc finger is necessary for the strong binding between SP1 and DNA. A crucial factor for nuclear localization is the coordinated binding of zinc fingers; removal of Sp1 from three zinc fingers eliminates both DNA binding and nuclear localization (Schröder, 1991; Kriwacki et al., 1992; Thiesen and; Ito et al., 2009; 2010). The N-terminus of the DNA domain comprises two adjacent trans-activating domains consisting of serine/threonine-and glutamine-rich regions. Between DNA and trans-activating domains is the C domain, a region with high charge. Zinc fingers can bind DNA without the C domain, but their ability to bind enhances with it.

**FIGURE 1:**
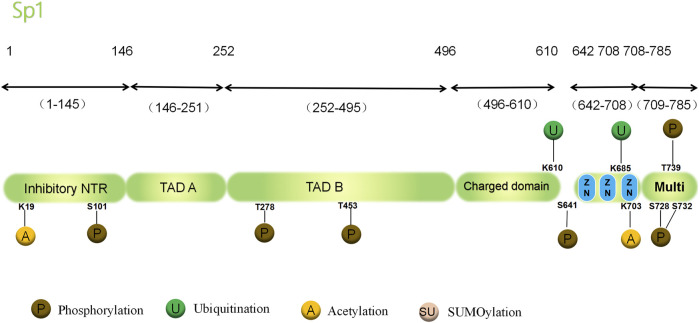
Structure and PTMs sites associated with cancer of the human Sp1 protein. PTM, post-translational modifications; Sp1, the transcription factor Specificity Protein 1; NTR, N-terminal region; TAD, Topologically Associating Domain; Multi, multimerization domain; S, Ser, serine; T, Thr, threonine; K, Lys,lysine.

Sp1 also contains a multimerization domain that can promote molecular interaction between unbound Sp1 and DNA, leading to the formation of tetramers. The tetramers progress to form larger polysomes, leading to the connection between the nearby promoter area and distant enhancer, the interaction of numerous Sp1 molecules, and the hyperactivation of genes (Mastrangelo et al., 1991; Beishline and Azizkhan-Clifford, 2015). In brief, Sp1’s structural region is comprised of three Cys2-His2 zinc finger domains, with its binding to the GC box controlling the basal and inducible gene transcription. However, the regulatory mechanisms vary among target genes and cells, involving direct interactions between proteins and DNA, interactions between proteins, and changes in chromatin structure due to changes in Sp1 expression or PTMs. In transcriptional regulation, PTMs, such as phosphorylation, acetylation, ubiquitylation, glycosylation, and small ubiquitin-related modifiers (SUMO), affect the activity and stability of Sp1. In reaction to various physiological and pathological stimuli, they control distinct gene programs and have the ability to alter the structure of Sp1 in diverse cells (Figure 1).

### 2.2 Function of Sp1 protein in cancer

Sp1 is the initial transcription factor for RNA polymerase II in mammals to be identified; it can participate in multiple biological processes, such as cell proliferation, apoptosis, differentiation, and transformation. Additionally, it is central to regulating transcription factor activity and cell signaling pathways. Sp1 is capable of not just sustaining basic transcription, but also controlling (inducing and inhibiting) the transcriptional function of various transcription factors (Narayan et al., 1997; Cawley et al., 2004; Chu, 2012; Gilmour et al., 2014). Sp1 has been recognized as a transcription factor with widespread importance, its activity is tightly controlled during tumorigenesis. Sp1 expresses strong pro-cancer functional activity and abnormal expression in numerous cancer types. Moreover, its levels are associated with the likelihood of survival for nearly all individuals diagnosed with cancer. High Sp1 levels often predict poor prognosis; therefore, researchers have identified Sp1 as a tumor marker (Hanahan and Weinberg, 2000; Maksimovic-Ivanic et al., 2012; Beishline and Azizkhan-Clifford, 2015).

Cancer is a complex disease and exhibits substantial differences between and within different tumor types. The heterogeneity among different cancer types makes treatment difficult (Safe et al., 2018). Nonetheless, commencing with relatively well-defined commonalities in cancers could serve as a starting point for creating better treatment plans and addressing challenges related to tumor diversity (Hanahan and Weinberg, 2011). Tumorigenesis is defined as uncontrolled cell cycle progression, with the phenotypic and genotypic differences partly correlated with the complex pathways leading to tumor development (Becker et al., 1981; Zhao et al., 2015). Fourteen major features of cancer, namely, the signals of continuous proliferation, replicative immortalization, resistance to cell death, immune escape, angiogenesis induction, cell invasion and metastasis, cellular energy dysregulation, inflammatory and unstable genome, unlocking phenotypic plasticity, nonmutational epigenetic reprogramming, polymorphic microbiomes, and senescent cells were identified in a systematic review of cell pathways involved in tumor formation and progression (Maksimovic-Ivanic et al., 2012; Hanahan and Weinberg, 2000; Hanahan. 2022).

The intricate functionality of Sp1 is evidenced by its ability to modulate key genes that impact the top ten characteristics of cancer (Beishline and Azizkhan-Clifford, 2015); It affects eight major features of cancer by regulating several key genes. For example, Sp1 can maintain the continuous tumor proliferation signal by mediating the transcription of epidermal growth factor (Pascall and Brown, 1997) and its receptor (Kageyama et al., 1988; Kitadai et al., 1992), fibroblast growth factor (Payson et al., 1998; Jimenez et al., 2004), insulin-like growth factor (Hyun et al., 1993; Jiang et al., 2004), and its receptor (Jensen et al., 1995; Ohlsson et al., 1998; Abramovitch et al., 2003; Maor et al., 2007). Sp1 enables the cancer cells to avoid senescence and achieve replication immortalization through the genes encoding the cyclin-dependent kinase (CDK) inhibitor p16 (Ink4a), p53, and various factors involved in maintaining telomeres, including telomerase. Furthermore, Sp1 can escape growth inhibition by regulating cancer cell response to specific stresses. By controlling various pro- and anti-apoptotic elements, it can develop a resistance to immune system signals that trigger cell death from the outside (Ohlsson et al., 1998; French and Tschopp, 2002). Furthermore, it has the ability to enhance tumor cell resistance to programmed cell death and evasion of the immune system. Sp1 activates the angiogenic pathway through regulating the vascular endothelial growth factor (Abdelrahim et al., 2004; Stoner et al., 2004; Yao et al., 2004; Abdelrahim and Safe, 2005; Yu et al., 2010; Eisermann et al., 2013), platelet reactive protein 1 (Okamoto et al., 2002), platelet-derived growth factor (Santiago and Khachigian, 2004), urokinase plasminogen activator (Trisciuoglio et al., 2004; Belaguli et al., 2010), and anti-angiogenic genes to meet the nutritional and oxygen requirements of tumor growth. Moreover, it triggers the invasion and spread of tumor cells by influencing various factors that promote or inhibit invasion, including matrix metalloproteinases (MMPs) (Ma et al., 2004; Hsu et al., 2006). MMP inhibitors reverse the induction of a cysteine-rich protein (RECK). Furthermore, Sp1 has also been shown to be associated with “contributing features” of cancer, namely, the inflammatory and unstable genome. It is noteworthy that Hanahan proposed four emerging hallmarks and enabling characteristics of cancers in 2022, based on previous research on 10 features. These include “unlocking phenotypic plasticity”, “nonmutational epigenetic reprogramming”, “polymorphic microbiomes”, and “senescent cells”, which he referred to as “trial balloons”. The specific relationship between Sp1 and these “trial balloons” necessitates further theoretical and clinical investigation (Hanahan. 2022).

There is a wealth of evidence indicating that various compounds, such as approved drugs for alternative medical conditions, have the capability to downregulate or degrade Sp1, thereby impeding cell/tumor proliferation and invasion and promoting apoptosis. Among these compounds are HDAC inhibitors, metformin, bardoxolone methyl, bortezomib, and select non-steroidal anti-inflammatory drugs (NSAIDs). Nevertheless, existing anticancer medications targeting Sp1 have yet to be integrated into standard cancer treatment regimens, necessitating further assessment of their clinical utility in combination therapies, including drug repurposing strategies (Safe et al., 2016; 2018; 2023).

Most of the mentioned functions are affected by PTMs, such as phosphorylation, ubiquitination, glycosylation, SUMOlytion, methylation, and acetylation (Jha et al., 2024). A substantial number of PTMs increases the number of proteins with underlying molecular states, expanding the protein diversity and facilitating the emergence of organismal complexity (Snider and Omary, 2014). PTMs occur more rapidly than protein synthesis; they can occur in different stages of the protein “life” cycle. PTMs are central to signal transduction and life processes; they enable cells or organisms to respond promptly according to the changes in the surrounding environment (Beltrao et al., 2012). Several studies have illustrated the anticancer effects of drugs through PTMs of Sp1. For instance, atractyloide-1 has been shown to impede the growth of lung cancer cells by activating ERK1/2-mediated Sp1 phosphorylation (Xiao et al., 2017). Thiazolidinedione derivatives (OSU-CG12) have demonstrated the ability to hinder prostate cancer progression by facilitating Sp1 degradation through modulation of Sp1 phosphorylation and ubiquitination levels (Wei et al., 2009). Similarly, the curcumin analog EF24 has been found to degrade Sp1 by enhancing its ubiquitination, thereby impeding Sp1-induced proliferation and invasion of TNBC cells and ultimately inhibiting TNBC advancement (Duan et al., 2022). Taken together, Sp1 PTMs are vital in tumorigenesis and progression. This review systematically reviews the regulatory effects of Sp1 PTMs on cancer to provide novel ideas and strategies for cancer treatment and prevention.

## 3 Sp1 PTMs in cancer

### 3.1 Phosphorylation

Phosphorylation is the core PTM in cellular signaling transduction; it is intimately involved in most cellular processes (Cohen, 2002). Protein phosphorylation cascades form the backbone of signaling pathways that can regulate substrate activity, stability, protein interactions, and subcellular localization. Phosphorylated Sp1 is central to the regulation of multiple cancer-related genes (Chu and Ferro, 2005; Shiloh and Ziv, 2013). Several key signaling kinases, including extracellular signal-regulated protein kinases (ERK) one and 2, ataxia-telangiectasia mutated (ATM) kinase, JNK1, CDK2, PI3K, and Rad 3-related kinase, phosphorylate Sp1 to control proliferation, movement, and invasion of tumor cell. The phosphorylation events play a role in further PTMs, regulating Sp1 stability, enhancing its DNA-binding affinity, and enabling transcriptional activation (Beishline and Azizkhan-Clifford, 2015).

#### 3.1.1 ERK-mediated Sp1 phosphorylation

The MAPK cascade plays a crucial role in controlling various cellular activities through signaling pathways. It activates and phosphorylates downstream proteins involved in cell proliferation, division, apoptosis, and reaction to stress (Guo et al., 2020). Of them, the Ras/Raf/MAPK/ERK pathway is an important signaling cascade in the MAPK signal transduction pathway; it is central to transmitting extracellular signals to intracellular targets (Plotnikov et al., 2011). Invasion and proliferation of tumor cells are mediated by activation of ERK kinase through Sp1 modification.

Inhibition of gene expression is caused by the interaction of histone deacetylases with Sp1 (Zhao et al., 2003). RECK, the matrix metalloproteinase (MMP) inhibitory protein, could potentially prevent tumor metastasis by controlling MMP activity in a negative manner. Hsu et al. (2006) demonstrated that in neuroblastoma (B104-1–1) cells, the proto-oncogene HER-2/neu could inhibit RECK (a tumor metastasis suppressor) level through suppressing ERK and Sp1 transcription factor; eventually, it promoted cell invasion. HER-2/neu promotes Sp1 phosphorylation at Thr453 and Thr739 and binds to the RECK promoter through the ERK protein kinase. As a result of phosphorylation of Sp1, histone deacetylase 1 (HDAC1) is recruited to the RECK promoter, suppressing RECK expression and facilitating invasion of cells.

Telomerase reverse transcriptase (TERT) is responsible for encoding the catalytic component of telomerase. Reacting the TERT is crucial for maintaining telomeres, and thus for supporting the uncontrolled division of cancer cells. Furthermore, it mediates tumorigenesis and progression. There is a high frequency of TERT promoter mutations in various cancer types that predict poor outcomes in patients. Specific mutations in key areas attract the GA Binding protein transcription factor α (GABPA), which in turn enhances TERT transcription (Bell et al., 2015). Mutations in the BRAF^V600E^ gene are frequently seen as a genetic change that promotes the development and advancement of tumors, especially in cases of thyroid cancer and melanoma. Wu et al. reported that in BRAF^V600E^-mediated human cancers, such as glioblastoma and thyroid cancer, the GABPA recombinant protein and Sp1 co-activate the mutated TERT promoter, accelerating cancer incidence and development (Batista et al., 2016; Wu et al., 2021). Briefly, in BRAF^V600E^-induced cancer cells, the GABP tetramer facilitates the activation of ERK-recruited mutated TERT promoters; ERK activation promotes the dissociation of HDAC1 from Sp1/HDAC1 complex by upregulating the phosphorylation levels at sites, such as Sp1 Thr 739. Thus, Sp1 and GABPA interaction is strengthened, and the binding between GABPA and the mutant TERT promoter is enhanced. The combined effect of Sp1 and GABPA sustains a dynamic chromatin state in the altered TERT promoter. Furthermore, it contributes to TERT reactivation, promoting cancer incidence and progression. Caveolin-1 (Cav-1), an essential protein found in a structural pit, acts as an oncogene in hepatocellular carcinoma (HCC) by contributing to abnormal glycosylation of proteins. Zhang et al. (2019) found that Cav-1 in the plasma membrane is implicated in facilitating the abnormal glycosylation of HCC invasion and metastasis by regulating β-1, 3-n-acetylglucosaminyltransferase (Rfng) expression; it plays a similar role in an oncogene. Furthermore, in mouse HCC cell lines, Cav-1 phosphorylated the transcription factors Hnf4a and Sp1 by activating the ERK-JNK-P38 pathway. In turn, it enhanced Rfng mRNA levels and protein expression by promoting Hnf4a and Sp1 binding to the Rfng promoter region. Finally, Cav-1 is involved in mediating HCC invasion and metastasis.

#### 3.1.2 ATM kinase-mediated Sp1 phosphorylation

Unrepaired severe DNA damage, termed double-strand breaks (DBS), disrupts DNA replication severely in proliferating cells. It leads to cell death or chromosome aberrations, triggering a vicious cycle of genomic changes and eventually inducing cancer (Shiloh and Ziv, 2013). ATM is the key mobilizer of cellular reaction to DNA impairment; Sp1 can contribute to DNA restore response and regulate cancer through ATM-mediated phosphorylation. Fletcher et al. (2018) demonstrated that when DNA is damaged, ATM-mediated Sp1 phosphorylation can downregulate the ability of DNA base removal repair (BER), the primary repair pathway of endogenous DNA damage, thus promoting cell apoptosis. During excessive DNA damage or defective DNA repair, activated ATM recognizes the break in the DNA strand and enhances the phosphorylation of the Sp1 at the Ser101 site to induce its degradation. However, degraded Sp1 can inhibit DNA repair by downregulating the level of the crucial base excision restore genes XRCC1 and DNA ligase III in the BER pathway, leading to DNA strand break accumulation and forming a vicious cycle. Epstein-Barr virus (EBV) is a carcinogenic herpes virus linked to human malignancy. The lytic reactivation of EBV induces ATM-dependent DNA damage in the cells with latent EBV infection. ATM activation triggers the transcription of viral lytic genes to stimulate lytic reactivation. Hau et al. (2015) reported that EBV lytic infection can trigger the activation of ATM kinase in nasopharyngeal epithelial cells. Activated ATM promotes EBV replication protein accumulation in the replication region by inducing EBV lysis infection and recruiting viral lytic proteins to the region. Of them, Sp1 phosphorylation is a crucial step following of ATM activation contributed to the forming of viral replication regions.

#### 3.1.3 PI3K kinase-mediated Sp1 phosphorylation

Apart from ERK and ATM kinases, PI3K can participate in cancer development by promoting Sp1 phosphorylation. Elevated luteinizing hormone (LH) has been associated with tumor development, including those found in the breasts and ovaries. Zhang et al. (2006) reported that trichostatin A (TSA) promotes LH receptor (LHR) gene level in human breast cancer cells (MCF-7) and human placental villous cells (JAR) by enhancing PI3K/PKC phosphorylation at Ser641, which finally promotes the release of suppressor protein p107 from the LHR promoter gene. Therefore, PI3K/PKC-mediated the phosphorylation of Sp1 promotes activating TSA-mediated LHR gene expression.

Immune escape plays a crucial role in the advancement of gastrointestinal solid tumors, with SP1 also playing a role in mediating this process (Zhou et al., 2021). In their study, Mao et al. (2023) demonstrated that Pleckstrin-2 in gastric cancer can enhance the expression of MT1-MMPs through the PI3K-AKT-Sp1 signaling pathway, resulting in the release of MICA and subsequently impairing NK cell-mediated immune surveillance, thereby facilitating tumor progression. Sp1 was identified as a key regulator of MT1-MMP expression and was shown to positively regulate its levels. The underlying mechanism involves Pleckstrin-2 activation of the PI3K/AKT pathway, leading to PI3K kinase-mediated phosphorylation of Sp1 at the T453 site, thereby increasing Sp1 expression and subsequently promoting MT1-MMP expression.

Some phosphorylation studies of Sp1 do not specify the kinases involved. Neovascularization, a critical process in the development and progression of tumors, plays a role in glioblastoma multiforme (GBM) basement membrane resistance to temozolomide (TMZ). Wang et al. (2024) discovered that in TMZ-resistant tumors, chemotherapy induces the phosphorylation of Sp1 at position S101, resulting in increased Sp1 activity and transcriptional upregulation of CCBE1. This upregulation of CCBE1 promotes the maturation of vascular endothelial growth factor C (VEGFC) and activates the VEGF receptor 2 (VEGFR2), VEGFR3, and Rho signaling pathways in vascular endothelial cells (VECs). Ultimately, this leads to increased angiogenesis in TMZ-resistant tumors, providing resistance to GBM cells in a VEC-dependent manner.

The promotion of tumor progression by SPNS2 is facilitated through the regulation of tumor immunity and the enhancement of tumor cell migration and invasion. Wang et al. (2023) utilized a transcription factor activity microarray to identify transcription factors that exhibit increased mRNA expression of SPNS2 in hepatocellular carcinoma (HCC) under conditions of iron deficiency. The findings indicated that iron deficiency specifically upregulated the transcriptional activity of HIF1α and Sp1 on SPNS2, with Sp1 demonstrating a more pronounced increase in transcriptional activity compared to HIF1α. The protein level of HIF1α was found to be elevated in cases of iron deficiency, while the protein level of Sp1 remained unchanged; however, the phosphorylation level of Sp1 showed an increase. Treatment with PX478, an inhibitor of HIF1α, and Mithramycin A, an inhibitor of Sp1, resulted in a reversal of the upregulation of SPNS2 mRNA and protein expression caused by iron deficiency, with Mithramycin A exhibiting a more pronounced effect. These findings suggest that Sp1 serves as a primary regulator of iron deficiency-induced SPNS2 expression, with phosphorylation playing a crucial role in this regulatory mechanism.

Taken together, a thorough understanding of Sp1 phosphorylation is important to explore Sp1 protein function and carcinogenesis mechanisms. Further studies may elucidate additional phosphorylation targets and their role in biological processes to precisely define the role of Sp1 phosphorylation in cancer. Furthermore, they may highlight novel targets for antitumor drug development and promote cancer prevention and treatment (Chu et al., 2005; Tan and Khachigian, 2009).

### 3.2 Ubiquitination

Ubiquitin modification conjugates polyubiquitin chains with target proteins to control the activeness, durability, and position of target proteins, thereby mediating proteasome degradation. Ubiquitin modification can regulate numerous cell activities, containing cell division, programmed cell death, regulation of gene expression, repairing damaged DNA, and the body’s defense mechanism (Mukhopadhyay and Riezman, 2007). The E1, E2, and E3 ubiquitin ligases are involved in Sp1 ubiquitination (Park et al., 2020). Several key signaling kinases, such as JP3, JP1, AKT, human leukocyte antigen complex 11 (HCG11), tripartite motif-containing protein 25 (TRIM25), RNF4, BAP1, FK506-binding protein 3 (FKBP3), RNF4, mediate Sp1 ubiquitination *in vitro* and *in vivo*. Other E3 ubiquitin ligases can regulate cancer through Sp1 ubiquitination. Sp1, a particular protein transcription factor, is found to be highly expressed in many tumors, serving as a gene that is not oncogenic but essential for cancer growth. Moreover, it acts as an unfavorable predictor for patient survival (Hedrick et al., 2016). Therefore, Sp1 degradation mediated by increased ubiquitination may inhibit cancer. The regulation of Sp1 ubiquitination has been linked to the advancement of various cancers such as gastric cancer (GC), melanoma, lung cancer, breast cancer, colorectal cancer, acute myeloid leukemia, and more.

Matrix metalloproteinase 2 (MMP2) is crucial in tumor-mediated extracellular matrix degradation. MMP2 overexpression can promote GC angiogenesis. According to Chen et al. (2014 and 2020), JWA, a tumor suppressor gene, could inhibit MMP2 mRNA levels and protein expression, thereby inhibiting GC angiogenesis. On the basis of the functional characteristics of JWA protein, Chen et al. (2020) designed MMP2-targeted JP3 anticancer peptides; it demonstrated therapeutic effects and no adverse effects in animal models. JP3 has a beneficial effect in preventing GC angiogenesis by affecting the TRIM25-SP1-MMP2 pathway. In GC cells, JP3 can increase TRIM25 stability and delay its degradation by promoting the phosphorylation of E3 ubiquitin ligase TRIM25 Ser12. Subsequently, TRIM25 promotes Sp1 degradation by ubiquitination at the K610 site, thereby reducing MMP2 transcription and translation. Finally, it leads to reduced angiogenesis and tumor proliferation in GC.

The presence of integrin αvβ3 is common on the outer layer of melanoma cells and has been linked to the growth of blood vessels in tumors, movement, growth and spread of cells (Huang and Rofstad, 2018). A functional polypeptide called JP1 was discovered by analyzing the JWA protein’s active portions (Cui et al., 2020). Micro-positron emission tomography suggests that JP1 targets melanoma cells and inhibits their growth and spread by inhibiting integrin αvβ3; moreover, JP1 prolongs the survival time in mice with melanoma. The specific mechanism involves targeting the JP1 peptide into melanoma cells highly expressing integrin αvβ3 and activating p-MEK1/2. Subsequently, p-MEK1/2 activates the E3 ubiquitin-ligase NEDD4L, which then enhances the ubiquitination of Sp1 at K685, accelerates Sp1 degradation, and suppresses integrin αvβ3-mediated transcription.

Ding et al. (2023) reported on substantially downregulated JWA protein levels in non-small cell lung cancer (NSCLC) associated with smoking, which was also connected to lower survival rates. JWA expression reduces in lung cancer cells exposed to nicotine in a manner that depends on the dosage. Nicotine decreases JWA levels via the AKT pathway mediated by CHRNA5. Subsequently, JWA inhibits transcription and translation by promoting ubiquitination-mediated clusters of differentiation (CD)44 degradation, eventually inhibiting lung cancer progression. Zhu et al. (2017) conducted *in vitro* and *in vivo* trials where FKBP3 enhanced the growth of NSCLC cells. Additionally, the overexpression of FKBP3 mRNA and protein was observed in NSCLC specimens, correlating with unfavorable outcomes in NSCLC patients. Elevated levels of FKBP3 substantially increased histone deacetylase 2 (HDAC2) expression and decreased cell cycle inhibitor p27 expression. Additionally, FKBP3 inhibited p27 levels by promoting HDAC2expression, thus inducing the proliferation of NSCLC cells. Through restraining Sp1 ubiquitination, FKBP3 activates the HDAC2 promoter. Subsequently, it promotes the binding between HDAC2 and p27 promoter, finally inhibiting p27 expression. Furthermore, miR-145-5p has the ability to attach to the 3′UTR of FKBP3, resulting in the suppression of FKBP3 gene expression. Taken together, miR-145-5p suppressed the growth of NSCLC by targeting the FKBP3/Sp1/HDAC2/p27 signaling pathway.

Similar processes occur in breast and colorectal cancers. Duan et al. (2022) demonstrated that treatment using curcumin analog (EF24) decreased tumor size, suppressed cell growth, and stimulated cell death in a mouse triple-negative breast cancer (TNBC) xenotransplantation model. Increased levels of HCG11 can suppress the ubiquitination of Sp1, leading to elevated Sp1 levels, which in turn promote survival and invasion of TNBC cells. Treatment with EF24 can inhibit HCG11 expression and promote the ubiquitination-mediated degradation of Sp1, thereby delaying Sp1-induced living and invasion of TNBC cells, ultimately inhibiting TNBC progression. According to Ren et al. (2023), long intergenic non-protein coding RNA955 (LINC00955) was downregulated in colorectal carcinoma and was linked to poor outcome. LINC00955 inhibits colorectal carcinoma cells growth both *in vivo* and *in vitro*. LINC00955 can interact with Sp1 from nucleotides 2073 to 2204 and with TRIM25 from nucleotides 984 to 1135, respectively. Therefore, LINC00955 may serve as a structural support for the interaction of TRIM25 and Sp1, facilitating their binding. The ubiquitin-ligase TRIM25 promoted Sp1 ubiquitination at K610 and its degradation. Moreover, it inhibited DNMT3B transcription and expression. Blocking DNMT3B leads to reduced methylation of the E3 ligase PhIP promoter, which increases PhIP transcription, promotes CDK2 ubiquitination and degradation, and finally leads to the growth arrest of colorectal cancer cells in the G0/G1 phase. Moreover, it inhibits the proliferation of colorectal cancer cells.

Fei et al. (2023) revealed that the deprivation of suppressor of DNA-binding-1 (ID1) in the bone marrow environment inhibited acute myeloid leukemia (AML) progression in a mouse model of AML. Co-culture of mesenchymal cells with AML cells showed significant reductions in Sp1 expression with ID1 absence. ID1 can compete with Sp1 for combining to the SIM domain of RNF4 to inhibit Sp1 degradation, thus promoting the transcription and translation of angiopoietin-like protein 7 (ANGPTL7) and AML progression. Cathepsin K displays high matrix degradation activity and is central to cancer invasion and progression. According to Seo et al. (2022), cathepsin K inhibitor odanacatib (ODN) can induce the deubiquitination enzyme BAP1 to phosphorylate at the Ser 592 site in human tumor cell lines (human renal clear cell carcinoma skin metastatic cells, human renal adenocarcinoma cells, human breast cancer cells, and human glioma cells). Moreover, ODN promotes its binding to Sp1, resulting in Sp1 deubiquitination and increased stability. Furthermore, it promoted SP1-mediated upregulation of bcl-2-associated X protein (Bax), thereby mediating oxaliplatin-induced apoptosis of human tumor cells. Consequently, Sp1 is the key transcription factor in ODN-mediated Bax enhancement, while BAP1 regulates Sp1 stability.

There is an association between cancer and abnormal actuation of Wnt signaling pathway as well as mutations of related regulatory factors. β-Catenin stabilization after the activation of Wnt signaling is a core event in this process. β-Catenin stability is controlled by a cytoplasmic destruction complex which contains scaffold proteins axin1, APC, glycogen synthase kinase 3β (GSK3β), and CSK1α (MacDonald et al., 2009; Fagotto, 2013). Without a Wnt ligand, β-catenin is attached to and undergoes phosphorylation by the disruption complex. The ubiquitin E3 ligase β-TrCP recognizes and degrades phosphorylated β-catenin according to MacDonald et al. (2009). Mir et al. (2018) reported elevated Sp1 expression in colorectal cancer cells driven by the Wnt signal; they also confirmed Sp1 was the direct target of the Wnt signaling pathway. Without Wnt signaling, GSK3β promotes Sp1 phosphorylation at S726 and S732 sites; it induces phosphorylated Sp1 degradation by E3 ubiquitin ligase β-TrCP-mediated ubiquitination. Upon activation of the Wnt signal, the interaction between Sp1 and β-catenin prevents the β-TrCP and the destruction complex axin1 from inducing the degradation of Sp1 ubiquitination. Therefore, Wnt signaling inhibits β-TrCP E3-mediated ubiquitination and subsequential degradation of Sp1. Additionally, Sp1 controls β-catenin stability by inhibiting its binding to the cytoplasmic destruction complex, suggesting a feedback regulatory mechanism between β-catenin and Sp1 in the Wnt signaling pathways. Sp1 not only stabilizes β-catenin but also regulates Wnt-responsive genes expression. By contrast, it binds to β-catenin and is necessary for its stable regulation in a Wnt-dependent manner. Sp1 and β-catenin combine in the nucleus to jointly control the expression of Wnt-responsive genes through sharing the promoter. Replenishing β-catenin levels in Sp1-deficient cells does not trigger the reactivation of Wnt-responsive genes. Taken together, Sp1 is necessary to stabilize β-catenin as well as to regulate Wnt response genes; therefore, it is a crucial factor in the molecular processes connected to the Wnt signaling pathway. Their mutual stabilization is crucial for target gene expression, which in turn impacts the advancement of cancer.

### 3.3 SUMOylation

SUMO proteins, which consist of four isoforms (SUMO1, SUMO2, and SUMO3, and SUMO4), are small eukaryotic protein modifiers that are conserved across species in the human genome. SUMO2 and SUMO3 have a similarity of up to 96%; thus, they are termed SUMO2/3. SUMO2/3 can form SUMO chains more efficiently than SUMO1. SUMO refers to a dynamic and reversible PTM process that binds SUMO protein to lysine residues covalently in the presence of E1, E2, and E3 ligase (Song et al., 2024). Proteins that are SUMOylated are subjected to degradation via the ubiquitin-proteasomes. SUMOylation and ubiquitination occur at identical proteins and lysine residues. Therefore, SUMOylation can compete against ubiquitination and antagonize ubiquitin-proteasomes-induced breakdown, potentially generating outcomes contrary to protein stability. SUMO-targeted E3 ubiquitin ligase (STUBL) is a new class of ubiquitin ligase, and RNF4 is the most studied human STUBL (Sriramachandran and Dohmen, 2014). SUMO proteases or the sentrin-specific protease (SENP) family can uncouple SUMO chains, reversing SUMOlation. SENP3 is specific for the SUMO 2/3 chain (Gong and Yeh, 2006). Wang et al. (2016) reported significantly increased SENP3 levels in GC cell lines, patients with GC, and nude mouse samples of GC. RNF4 recognizes SUMO2/3 conjugates of Sp1 and acts as a SUMO2/3 target of Sp1 ubiquitin E3 ligase. SENP3 can facilitate the uncoupling of Sp1 and SUMO2/3. Moreover, it can antagonize the degradation of Sp1 ubiquitination through eliminating the connection between Sp1 and RNF4, thus increasing the Sp1 protein level. Taken together, SENP3 enhances the proliferation of GC cells through antagonizing SUMO2/3-directed ubiquitination and preventing RNF4-induced proteasomal degradation, leading to elevated Sp1 protein expression.

Sp1 SUMOylation is also involved in the resistance of some chemotherapy drugs. Huang et al. (2022) revealed that in the tumor tissues of recurrent gastric cancer (GC) patients undergoing treatment with capecitabine and cisplatin, there was a decrease in Sp1 SUMOylation levels, an increase in Sp1 expression, and enhanced binding of Sp1 to the SNHG17 promoter. Additionally, SNHG17 was found to bind to miR-23b-3p and suppress its levels, while miR-23b-3p targeted Notch2. These findings suggest that reduced Sp1 SUMOylation leads to increased SNHG17 expression, thereby inhibiting the Notch2 suppression induced by miR-23b-3p and contributing to GC resistance to cisplatin. Furthermore, silencing Sp1 was shown to mitigate GC resistance to cisplatin. This study offers valuable insights for the development of potential therapeutic strategies to overcome chemoresistance in GC.

### 3.4 Glycosylation

Glycosylation is an enzymatic process characterized by glycosidic bond formation of sugars with other proteins sugars, or lipids (Moremen et al., 2012; Pinho and Reis, 2015). In most cases, glycosylation occurs on an o-linked chain and on an n-linked chain. Predominant sugar proteins consist of only one type of glycosylation; however, some protein polypeptides comprise both N-sugar and O-sugar chains. Additionally, certain proteins exhibit unique forms of glycosylation (Pinho and Reis, 2015). O-linked N-Acetylglucosaminylation (O-GlcNAcylation) refers to the addition of β-D-n-acetylglucosaminylation to serine or threonine residues of nuclear and cytoplasmic proteins; the disruption of its homeostasis has been contributed to cancer pathogenesis (Slawson and Hart, 2011; Yi et al., 2012; Yang and Qian, 2017). The O-GlcNAcylation of Sp1 modulates its nuclear localization, trans-activation, and stability (Han and Kudlow, 1997; Yang et al., 2001; Majumdar et al., 2006; Pinho and Reis, 2015).

Glutamine and lipids are the key components of cancer cell growth (Ryczko et al., 2016). It was demonstrated that when insulin and glutamine are lacking, the lipogenic transcription factor SREBP1 is activated in liver and breast cancer cells, which promotes its trans-activation and increases transcription by binding to glutamine synthetase (GS) promoters (Jhu et al., 2021). However, GS induced the expression of SREBP1/Acetyl Coenzyme a Carboxylase 1 (ACC1) by enhancing the Sp1 O-GlcNAcylation, leading to lipid droplet (LD) accumulation after insulin treatment. Additionally, the absence of glutamine triggers the creation of lipid droplets by activating the GS-controlled O-GlcNAc-Sp1/SREBP1/ACC1 pathway, which ultimately promotes the survival of cells. Hence, insulin and glutamine deprivation induce SREBP1-mediated transcriptional activation of GS, leading to Sp1 O-GlcNAcylation. Following this, O-GlcNAc-Sp1 enhances the transcription of SREBP1, creating a cycle of reinforcement that boosts the production of lipids and the formation of lipid droplets in both liver and breast cancer cells.

### 3.5 Acetylation

Acetylation of proteins is the predominant form of acylation alteration. Acetylation occurs through transferring the acetyl group from acetyl-CoA to the ε-amino side chain of lysine by lysine acetylase (KAT). Lysine acetylation refers to a dynamic post-translational reversible modification process. Most KATs can be classified into three families as follows: GCN5, p300, and MYST19. Deacetylation is catalyzed by deacetylases, such as NAD + -dependent sirtuin and Zn^2+^-dependent KDAC/HDACs. Acetylation impacts protein functionality by influencing various processes such as controlling protein stability, enzyme function, cellular positioning, and interactions with other PTMs. Both acetylation and deacetylation play crucial roles in regulating autophagy initiation and selective autophagy by modulating the acetylation levels of key proteins involved in the autophagy process. Additionally, these processes can influence gene expression by altering protein function. Acetylation of Sp1 can impact its DNA binding capacity and subsequent transcriptional regulation, thereby influencing cancer progression (Wang and Wang, 2019).

Histone deacetylase (HDAC), a type of protease, is crucial in modifying chromosome structure and regulating gene expression, thereby influencing the development of various tumors including lung cancer, breast cancer, prostate cancer, and gastrointestinal cancer. The relationship between HDAC2, HDAC10, and Sp1 acetylation in lung cancer progression is characterized by an upstream and downstream dynamic. For instance, in lung cancer, Sp1 acetylation is implicated in tumor macrophage polarization. Zheng et al. (2023) demonstrated that the close proximity of HDAC2-overexpressing M2-like tumor-associated macrophages to cancer cells is significantly associated with poor overall survival in lung cancer patients. Additionally, HDAC2 regulates the M2-like tumor-associated macrophages phenotype through acetylation of histone H3 and transcription factor Sp1. HDAC10 has been associated with a poor prognosis in patients with non-small cell lung cancer (NSCLC). To investigate the precise regulatory role and mechanism of HDAC10 in NSCLC, Guo et al. (2023) conducted experiments in which HDAC10 was knocked down in A549 and H1299 cells. The findings revealed that the knockout of HDAC10 suppressed the proliferation of NSCLC cells and induced ferroptosis by modulating the SP1/POLE2 axis. Specifically, HDAC10 knockout led to a reduction in Sp1 levels by promoting acetylation of Sp1 at the K703 site, thereby inhibiting the binding of Sp1 to the POLE2 promoter and decreasing the expression of POLE2. The effects of HDAC10 loss on NSCLC cell proliferation and ferroptosis were partially reversed by overexpression of Sp1 or POLE2.

Furthermore, Sp1 acetylation has been found to play a role in the regulation of cancers such as glioblastoma and pancreatic cancer. Glioblastoma, characterized as the most aggressive form of brain tumor with a poor prognosis and propensity for metastasis to adjacent healthy brain tissue, was the focus of a study by Liu et al. (2019) investigating the molecular mechanisms and pathological significance of bradykinin receptors in this cancer. Specifically, Liu et al. (2019) examined the expression of the two primary bradykinin receptors, B1R and B2R, in two human GBM cell lines, U87 and GBM8901. The findings indicated that bradykinin modulates the expression of IL-8 and the migration of GBM cells through the activation of STAT3 and Sp1. And the study revealed a positive correlation between high expression levels of STAT3 and Sp1 and the clinicopathological grade of glioma. Additionally, it was found that the activation of Sp1 was facilitated by an increase in acetylation modification of Sp1. Murthy et al. (2024) demonstrated that acetate derived from cancer-associated fibroblasts can drive the progression of pancreatic cancer by modulating polyamine metabolism through the ACSS2-SP1-SAT1 axis. Specifically, acetylation of Sp1 at K19 site, mediated by acetate/ACSS2, plays a crucial role in enhancing the stability and transcriptional activity of Sp1 protein, ultimately leading to increased SAT1 levels in an acidic environment.

### 3.6 Methylation

DNA methylation is a common occurrence in cancer, particularly in the initial phases of tumor development, and serves as a crucial mechanism for the epigenetic control of gene expression. This process is distinguished by widespread hypomethylation of the genome alongside localized hypermethylation of multiple 5′-cytosine-phosphate-guanine-3’ (CpG) islands, frequently encompassing gene promoters and initial exons (Locke et al., 2019). Jumaniyazova et al. (2024) investigated the methylation patterns of CDKN1, CDKN2A, MYC, SMAD3, Sp1, and UBC genes in the tumor tissues of 50 HPV-negative HNSCC patients. The findings indicated a significant elevation in Sp1 gene methylation within tumor tissue DNA as opposed to normal control tissue. Elevated methylation of Sp1 inhibited the expression level of Sp1 and subsequently inhibited the proliferation of HNSCC cells, suggesting that Sp1 methylation might as a molecular indicator of malignant cell growth.

### 3.7 Crosstalk between different PTMs

Waby et al. (2008) systematically summarized the PTMs of Sp family transcription factors. They elucidated a possible synergistic effect between different PTMs, thereby suggesting combinations, such as phosphorylation, acetylation, and SUMOlation can produce subtle changes in transcriptional activation. Multiple Sp1 PTMs, such as phosphorylation and ubiquitination, phosphorylation and acetylation, SUMO, and ubiquitination, can co-regulate a particular cancer type.

#### 3.7.1 Phosphorylation and ubiquitination

Wei et al. (2009) reported that thiazolidinedione derivatives (OSU-CG12) could induce Sp1 degradation in prostate cancer, similar to the effects of glucose deprivation. Moreover, OSU-CG12 could promote ERK-mediated phosphorylation of Sp1 at Thr739 and GSK3β-mediated phosphorylation of Sp1 at Ser728 and Ser732 sites. Subsequently, E3 ubiquitin ligase beta transducing protein repeat sequence protein (β-TrCP) recognizes phosphorylated Sp1 and promotes its ubiquitination degradation. Thus, OSU-CG12 mimics glucose starvation activation of β-TrCP-mediated degradation of Sp1 ubiquitination. By contrast, ERKs and GSK3β kinase-mediated phosphorylation are central to β-TrCP-mediated recognition of Sp1. Mir et al. (2018) demonstrated that GSK3β can promote Sp1 phosphorylation at S726 and S732 sites in colorectal cancer cell lines lacking Wnt signaling. Additionally, it induces the degradation of phosphorylated Sp1 by β-TrCP-mediated ubiquitination. Therefore, the synergistic effect of Sp1 phosphorylation and ubiquitination mediates its degradation, which has the eventuality of transformation to promote innovative proposals for treatment and safeguard against cancer.

Apart from the synergies of Sp1 phosphorylation and ubiquitination mediating its degradation, Sp1 phosphorylation protects against proteasome degradation. Wang et al. (2023) reported on upregulated signaling regulatory protein α (SIRPA) in osteosarcoma (OS) tissues, especially in metastatic tissues; moreover, it was linked to a negative clinical outlook. SIRPA knockdown reduces Sp1 stability and arginine uptake to disrupt OS cell migration. Briefly, SIRPA activates Sp1 phosphorylation at Thr278 through ERK to prevent RNF4-mediated ubiquitination, thereby promoting Sp1 stability. By binding with the SLC7A3 promoter, Sp1 can increase SLC7A3 transcription and translation levels and arginine uptake capacity, thereby facilitating epithelial-mesenchymal transition and metastasis of OS cells. Moreover, arginine is able to enhance Sp1 stability without relying on ERK, resulting in the creation of the “Sp1 stability cycle.” Therefore, the increase in SIRPA expression enhances the spread of osteosarcoma by activating the “Sp1 stable cycle” and facilitating arginine absorption through SLC7A3, making it a promising target for osteosarcoma treatment.

Chuang et al. (2008) reported on phosphorylated Sp1 in the mitosis stage of human cervical adenocarcinoma HeLa cells, breast cancer MDA-MB-231 cells, lung adenocarcinoma A549 cell epithelial tumor cell line, and rat glioma cell line. JNK1 was activated in HeLa cell mitosis and in breast tumors induced by N-methyl-N-nitrosourea. It increased Sp1 stability by phosphorylating Sp1 at Thr278 and Thr739. Additionally, JNK1 makes Sp1 immune to ubiquitin-dependent degradation, leading to its accumulation. Mutation at Thr278/739 in Sp1 leads to instability during mitosis, reduced transcriptional activity of 12(S) -lipoxygenase expression, and slower cell proliferation. Taken together, the activation of JNK1 is essential for the phosphorylation of Sp1 and for shielding against degradation through the ubiquitin-dependent pathway in tumor cell lines during mitosis.

#### 3.7.2 Phosphorylation and acetylation

Toll-like receptor 5 (TLR5) identifies the flagellin of Gram-negative and Gram-positive bacteria and performs multiple functions by activating the intracellular signaling pathways (Carvalho et al., 2012). The presence of TLR5 in the intestinal epithelium provides protection against colon cancer (Vijay-Kumar et al., 2008a). Flagellin has been suggested by researchers as a supplementary treatment for colon cancer due to its ability to enhance the effectiveness of radiation and chemotherapy, while also safeguarding healthy tissue from the negative impacts of these treatments (Vijay-Kumar et al., 2008b). Thakur et al. (2016) reported that butyrate, a metabolic product in the gut, can upregulate TLR5 in intestinal epithelial cells and enhance flagellin-induced immune response. In human colon carcinoma HT29 cells, butyrate dephosphorylates and acetylates Sp1 through Ser/Thr phosphatases, resulting in reduced binding between Sp1 and TLR5 promoter.

Radiotherapy combined with Temozolomide (TMZ) improves outcomes in patients with malignant glioblastoma; however, O6-methylguanine-DNA methyltransferase (MGMT)-mediated DNA restore causes TMZ resistance. Yang et al. (2019) demonstrated that the neurosteroid dehydroepiandrosterone (DHEA), a neurosteroid responsible for the wellbeing of neurons and astrocytes, can boost DNA repair through the stimulation of Sp1 phosphorylation and deacetylation. Additionally, DHEA can alleviate TMZ-induced DNA damage, thereby reducing its toxicity to glioblastoma cells. DHEA activates Sp1 via the LYN/AKT pathway. Subsequently, it phosphorylates Sp1 in TMZ-damaged DNA to achieve deacetylation by recruiting histone deacetylase HDAC1/2. Deacetylated Sp1 regulates DNA repair by binding to anti-proliferating cell nuclear antigens. Hence, Sp1 phosphorylation and deacetylation are central to DHEA-mediated DNA repair inducing TMZ resistance in glioblastoma.

## 4 Conclusion and perspective

**FIGURE 2:**
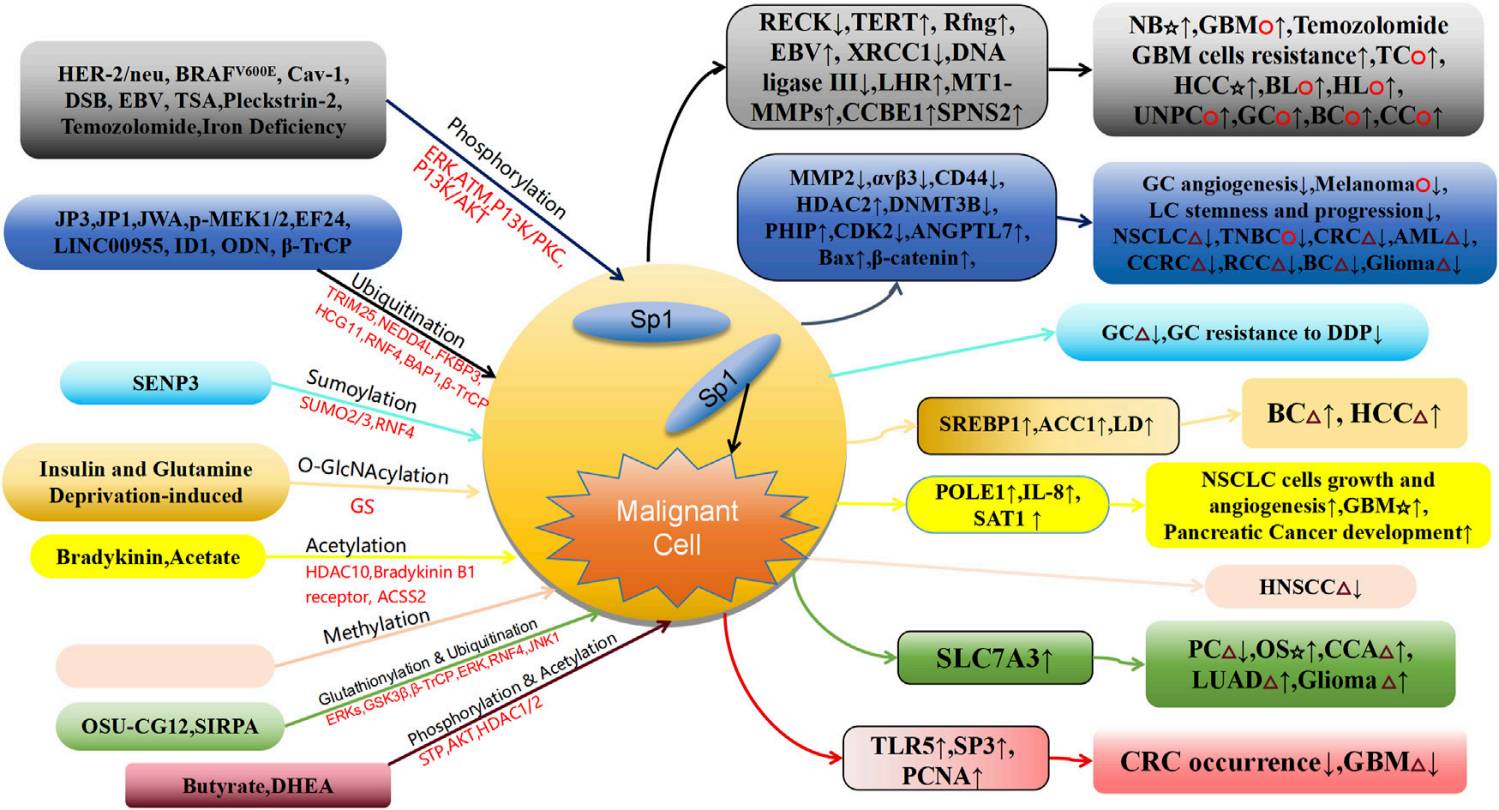
Schematic association of PTMs of Sp1 linked to cancer diseases. GC, Gastric Cancer; LC, lung cancer; NSCLC, Non Small-cell Lung Cancer; TNBC, Triple-Negative Breast Cancer; CRC, Colorectal Carcinoma; AML, Acute myeloid leukemia; CCRC, Clear Cell Renal Carcinoma; RCC, Renal Cell Carcinoma; BC, Breast Cancer; HCC, Hepatocellular Carcinoma; BL, Burkitt Lymphoma; HL, Hodgkin Lymphoma; UNPC, Undifferentiated Nasopharyngeal Carcinoma; CC, Choriocarcinoma; PC, Prostate Cancer; OS, Osteosarcoma; CCA, Cervical Cancer; LUAD, Lung Adenocarcinoma; GBM, Glioblastoma Multiforme; Cav-1, Caveolin-1; DSB, DNA Strand Breakage; LHR, luteinizing hormone receptor; TSA, Trichostatin A; LINC00955, Long Intergenic Non-protein Coding RNA955.☆, Tumor cell invasion and metastasis; △, Proliferation of tumor cells; ○, The proliferation, invasion and metastasis of tumor cells.

This article primarily summarizes the mechanism by which PTMs, such as phosphorylation, ubiquitylation, ubiquylation, SUMO, glycosylation, and acetylation regulate Sp1 stability and its regulatory role in cancer development (Table 1; Figure 2). For example, ERK kinase-mediated Sp1 phosphorylation cooperates with HDAC1 to inhibit RECK expression, promoting neuroblastoma invasion, and with GABPA to improve TERT transcription, promoting the growth and advancement of thyroid cancer (Hsu et al., 2006; Wu et al., 2021). Additionally, it cooperates with Hnf4a to promote Rfng transcription, mediating HCC invasion and metastasis (Zhang et al., 2019). ATM kinase-mediated Sp1 phosphorylation suppresses the repair pathway of DNA damage involving cancer development and induces EBV lytic infection to promote malignancy development (Fletcher et al., 2018). Additionally, ubiquitylation-mediated Sp1 degradation regulates tumor vascular angiogenesis and tumor cell proliferation by TRIM25-Sp1-MMP2 (Chen et al., 2020) and p-MEK1/2-NEDD4L-Sp1-αvβ3 pathway (Cui et al., 2020) and lung cancer progression by the CHRNA5-AKT-JWA-Sp1-CD44 pathway (Ding et al., 2023), is involved in NSCLC proliferation by the miR-145-5p-FKBP3/Sp1/HDAC2/p27 (Zhu et al., 2017) and colorectal cancer proliferation by LINC00955-TRIM25-Sp1-DNMT3B-PhIP-CDK2 pathway (Ren et al., 2023), promotes AML progression by the ID1-RNF4-Sp1-ANGPTL7 pathway (Fei et al., 2023), and is involved in ODN promoting oxaliplatin-induced apoptosis in human tumor cells by the BAP1-Sp1-Bax pathway (Seo et al., 2022). Thus, Sp1 PTMs are central to the initiation and progression of multiple cancers. Studies of Sp1 PTMs can facilitate the development of novel drug targets and biomarkers and offer important implications for the control of cancer.

**TABLE 1:** The PTMs of Sp1 in cancer.

Modification	Kinase	Materials	Sites	Function	Sp1 activity/Expression	Effect of Sp1 PTMs on cancers prognosis	References
Phosphorylation	ERK	HER-2/neu	Thr453 Thr739	RECK↓	Sp1↑	NB cells invasion↑	Hsu et al. (2006)
	ERK	BRAF^V600E^	Thr739	TERT↑	Sp1↑	GBM and TC cells proliferation and invasion↑	Wu et al. (2021)
	ERK	Caveolin-1	_	Rfng↑	Sp1↑	HCC cell proliferation and invasion↑	Zhang et al. (2019)
	ATM	DSB	Ser101	XRCC1↓ DNA ligase III↓	Sp1↓	Pre-cancerous cells↓	Fletcher et al. (2018)
	ATM	EBV	Ser101	EBV↑	Sp1↑	EBV replication↑, BL, HL, UNPC, GC↑	Hau et al. (2015)
	PI3K/PKCζ	TSA	Ser641	LHR↑	Sp1↑	BC and CC progression↑	Zhang et al. (2006)
	PI3K/AKT	Pleckstrin-2	Thr453	MT1-MMPs↑	Sp1↑	Immune escape of GC↑	Mao et al. (2023)
	_	Temozolomide	Ser101	CCBE1↑	Sp1↑	Temozolomide GBM cells resistance↑	Wang et al. (2024)
	_	Iron Deficiency	_	SPNS2↑	Sp1↑	HCC tumor cells migration and invasion↑	Wang et al. (2023)
Ubiquitination	TRIM25	JP3	K610	MMP2↓	Sp1↓	GC angiogenesis↓	Chen et al. (2020)
	NEDD4L	JP1, p-MEK1/2	K685	αvβ3↓	Sp1↓	Melanoma cells proliferation and invasion↓	Cui et al. (2020)
	PI3K/AKT	JWA	_	CD44↓	Sp1↓	Lung cancer stemness and progression↓	Ding et al. (2023)
	FKBP3	_	_	HDAC2↓	Sp1↓	NSCLC cells proliferation↓	Zhu et al. (2017)
	HCG11	EF24	_	_	Sp1↓	TNBC cells proliferation and invasion↓	Duan et al. (2022)
	TRIM25	LINC00955	K610	DNMT3B↓ PHIP↑ CDK2↓	Sp1↓	CRC cells proliferation↓	Ren et al. (2023)
	RNF4	ID1	_	ANGPTL7↓	Sp1↓	AML cells proliferation↓	Fei et al. (2023)
	BAP1	ODN	_	Bax↓	Sp1↓	CCRC, RCC, BC, Glioma cell proliferation↓	Seo et al. (2022)
	β-TrCP	Wnt	_	β-catenin↓	Sp1↓	CRC cells proliferation↓	Mir et al. (2018)
SUMOylation	SUMO2/3 RNF4	SENP3	_	_	Sp1↓	GC cells proliferation↓	Wang et al. (2016)
	_	_	_	SNHG17↓	Sp1↓	GC resistance to DDP↓	Huang et al. (2022)
O-GlcNAcylation	GS	Insulin and Glutamine Deprivation-induced	_	SREBP1↑ ACC1↑LD↑	Sp1↑	BC, HCC cells proliferation↑	Jhu et al. (2021)
Acetylation	HDAC10	_	K703	POLE1↑	Sp1↑	NSCLC cells growth and angiogenesis↑	Guo et al. (2023)
	Bradykinin B1 receptor	Bradykinin	_	IL-8↑	Sp1↑	GBM cells invasion↑	Liu et al. (2019)
	ACSS2	Acetate	K19	SAT1↑	Sp1↑	Pancreatic Cancer development↑	Murthy et al. (2024)
Methylation	_	_	_	_	Sp1↓	HNSCC cells proliferation↓	Jumaniyazova et al. (2024)
Glutathionylation and Ubiquitination	ERKs GSK3β β-TrCP	OSU-CG12	Thr739 Ser728 Ser732	_	Sp1↓	PC progression↓	Wei et al. (2009)
	ERK,RNF4	SIRPA	Thr278	SLC7A3↑	Sp1↑	OS cells invasion↑	Wang et al. (2023)
	JNK1	_	Thr278 Thr739	_	Sp1↑	CCA, LUAD Glioma cell proliferation↑	Chuang et al. (2008)
Phosphorylation and Acetylation	STP	Butyrate	_	TLR5↑SP3↑	Sp1↓	CRC occurrence↓	Thakur et al. (2016)
	AKT HDAC1/2	DHEA	_	PCNA↑	Sp1↑	GBM cells proliferation↑	Yang et al. (2019)

ERK, extracellular signal-regulated kinase; RECK, reversion inducing cysteine rich protein with kazal motifs; TERT, human telomerase reverse transcriptase; Rfng, β-1,3-N-acetylglucosaminyltransferase; ATM, ataxia telangiectasia mutated; DSB, DNA Strand Breakage; XRCC1, X-ray Repair Cross Complementing 1; EBV, Epstein-Barr virus; PKC, protein kinase C; TSA, Trichostatin A; LHR, luteinizing hormone receptor; LH, luteinizing hormone; TRIM25, tripartite motif-containing 25; JP3, an MMP2-targeted peptide, junctophilin-3; MMP2, Matrix Metalloproteinase 2; avβ3, integrinavβ3; PI3K/AKT, Phosphatidylinositol-4,5-bisphosphate 3-kinase/Akt; JWA, ADP ribosylation factor like GTPase 6 interacting protein 5 (ARL6IP5); EF24, a curcumin analog; LINC00955, Long Intergenic Non-protein Coding RNA 955; EF24, (3E,5E)-3,5-bis(4-fluorobenzylidene)piperidin-4-one; DNMT3B, DNA methyltransferase 3 beta; ID1, DNA binding 1; RNF4, an E3 ubiquitin ligase; β-TrCP, an E3 ubiquitin ligase; FKBP3, a member of FK506-binding proteins; HDAC2, histone deacetylase 2; ODN, odanacatib; DDP, cisplatin; GS, glutamine synthetase; SREBP1, sterol regulatory element-binding protein 1; ACC1, acetyl-CoA carboxylase 1; LD, lipid droplet; HDAC10, histone deacetylase 10; IL-8, Interleukin-8; ACSS2, Acyl-CoA synthetase short-chain family member 2; SAT1, Recombinant Human Spermidine/Spermine N1-Acetyltransferase 1; OSU-CG12, (Z)-5-(4hydroxy-3-trifluoromethylbenzylidene)-3-(1-methylcyclohexyl)thiazolidine-2,4-dione, a thiazolidinedione derivative; GSK3β: glycogen synthase kinase 3β; β-TrCP, β-transducin repeat-containing protein; SIRPA: signal regulatory protein alpha SLC7A3: solute carrier family 7 member 3; JNK1: c-Jun NH2-terminal kinase 1; DHEA: dehydroepiandrosterone ; PCNA: proliferating cell nuclear antigen; NB: neuroblastoma; GBM: glioblastoma multiforme; TC: thyroid carcinoma; HCC: hepatocellular carcinoma; BL: burkitt lymphoma; HL: hodgkin lymphoma; UNPC: undifferentiated nasopharyngeal carcinoma; GC: gastric cancer; BC: breast cancer; CC: choriocarcinoma; NSCLC: non small-cell lung cancer; TNBC: triple-negative breast cancer; CRC: colorectal carcinoma; AML: acute myeloid leukemia; CCRC: clear cell renal carcinoma; RCC: renal cell carcinoma; HCC: hepatocellular carcinoma; HNSCC, head and neck squamous cell cancer; PC, prostate cancer; OS, osteosarcoma; CCA, cervical cancer; LUAD, lung adenocarcinoma.

However, the mechanism behind drug-induced Sp1 downregulation is complex, and no anticancer drugs targeting Sp1 have been developed for clinical application. Various pathways, such as proteasome-mediated degradation, responses induced by cannabinoid receptors, zinc depletion, and the kinase/phosphatase pathway, can regulate the degradation of Sp1 (Safe et al., 2016; 2018; 2023). However, Sp1 PTMs control the transcription of many genes related to cancer hallmarks. Often, they serve as a key link; thus, Sp1 PTMs can be targeted for therapy in cancer. Researchers should consider the following aspects during anticancer drug development for Sp1 PTMs: First, Sp1 phosphorylation and ubiquitination encompass most cancer types, and the major pathways, and display more diverse functions; particularly, ubiquitylation modification is directly related to Sp1 degradation. Therefore, phosphorylation and ubiquitination can be used as the key aspects of drug development. Second, in Sp1 PTMs, the development of sites with high-frequency potentials, such as Thr278, Thr739, Ser101, and K610, and that of target drugs may increase the implication of treating multiple cancer types. Third, the transformation of normal cells to cancer cells has been linked to Sp1, Sp3, and Sp4, which display high expression in cancer cells. SP3 and SP4 have oncogenic functions; therefore, Sp1 can be considered as the key evaluation index rather than the sole efficacy evaluation index. Fourth, drug combination with chemotherapy can improve the therapeutic effects and overcome the resistance to cancer chemotherapy. The PTMs of Sp1 are also implicated in drug resistance to chemotherapeutic agents like Temozolomide and cisplatin, suggesting a potential avenue for overcoming resistance mechanisms. Certain types of small drugs used to treat cancer, like bortezomib, and drugs that help prevent cancer, such as sulindac, isothiocyanate, and polyphenols, can reduce Sp1 expression in cancer cells. The intention behind the development of these compounds was not to inhibit Sp1 or Sp1 regulatory genes, but it is possible that this activity is partly responsible for in their effectiveness as anticancer medications (Jia et al., 2010; Gao et al., 2011; Safe et al., 2018). Finally, owing to the complex functional role of Sp1, clinicians should consider the interaction among Sp1 PTMs, despite the need for synergistic initiation in the regulation of a particular cancer or identical cancer-related genes.
